# A cluster randomized controlled trial of a behavioral intervention to facilitate the development and implementation of clinical practice guidelines in Latin American maternity hospitals: the Guidelines Trial: Study protocol [ISRCTN82417627]

**DOI:** 10.1186/1472-6874-5-4

**Published:** 2005-04-11

**Authors:** Fernando Althabe, Pierre Buekens, Eduardo Bergel, José M Belizán, Nora Kropp, Linda Wright, Norman Goco, Nancy Moss, for the Guidelines Trial Group

**Affiliations:** 1 Perinatal Research Unit (PRU), Montevideo, Uruguay; 2 School of Public Health and Tropical Medicine, Tulane University, New Orleans, Louisiana, USA; 3 Institute of Clinical Effectiveness and Health Policy (IECS), Buenos Aires, Argentina; 4 RTI International, North Carolina, USA; 5 Center for Research for Mothers and Children, National Institute of Child Health & Human Development, USA

## Abstract

**Background:**

A significant proportion of the health care administered to women in Latin American maternity hospitals during labor and delivery has been demonstrated to be ineffective or harmful, whereas effective interventions remain underutilized. The routine use of episiotomies and the failure to use active management of the third stage of labor are good examples.

**Methods/Design:**

The aim of this trial is to evaluate the effect of a multifaceted behavioral intervention on the use of two evidence-based birth practices, the selective use of episiotomies and active management of the third stage of labor (injection of 10 International Units of oxytocin). The intervention is based on behavioral and organizational change theories and was based on formative research. Twenty-four hospitals in three urban districts of Argentina and Uruguay will be randomized. Opinion leaders in the 12 intervention hospitals will be identified and trained to develop and implement evidence-based guidelines. They will then disseminate the guidelines using a multifaceted approach including academic detailing, reminders, and feedback on utilization rates. The 12 hospitals in the control group will continue with their standard in-service training activities. The main outcomes to be assessed are the rates of episiotomy and oxytocin use during the third stage of labor. Secondary outcomes will be perineal sutures, postpartum hemorrhages, and birth attendants' opinions.

## Background

In Latin American maternity hospitals, a significant proportion of the health care administered to women during labor and delivery has been demonstrated to be ineffective or harmful, whereas proven effective interventions are underutilized. For example, routine episiotomy use has been documented to be useless and even harmful [1], yet the episiotomy rate in vaginal births of primiparous women in Latin American hospitals is 92 %. [2]. The caesarean section rate in Latin American countries is above 25% for the region as a whole, resulting in approximately 850,000 unnecessary cesarean sections performed each year [3]. Simultaneously, many of the birth practices verified as beneficial are not routinely used, e.g. active management of the third stage of labor has been proven effective to prevent postpartum hemorrhage, one of the leading causes of maternal deaths in the developing world [4]. In a survey in 19 maternity services of Uruguay and Argentina, 17 (89.5%) used expectant management as the standard form of care (unpublished observation). Another practice that has been proven to be beneficial and without adverse effects is a companion accompanying women in labor and delivery [5]; however, only 21% of women had a companion in the survey of the 19 hospitals mentioned above. The administration of enemas at admission in labor, perineal shaving, and systematic intravenous infusion are other examples of practices not supported by scientific evidence which are still in widespread use in Latin American hospitals [6,7].

### Barriers to the adoption of evidence based birth practices

Why, despite scientific evidence and active dissemination of scientific information, are harmful or unnecessary procedures (or both) still used, whereas other beneficial procedures are ignored?

It is postulated that a wide variety of barriers can hinder practitioners from adhering to evidence-based practices, thus a theoretical framework may help delineate these barriers and possibly help target specific interventions to address appropriate practice patterns. Cabana et al. published a comprehensive systematic review about the barriers to physician adherence to practice guidelines and developed a theoretical approach to the barriers [8].

They reviewed 76 articles including five qualitative studies and data from 120 surveys to identify possible barriers to guideline adherence. Barriers were classified into seven general categories: barriers affecting physician knowledge (lack of awareness and lack of familiarity); those affecting attitudes (lack of agreement, lack of self-efficacy, lack of outcome expectancy and the inertia of previous practices); and those affecting behavior (external barriers).

One study included in Cabana's review was a survey of 38 childbirth-related organizations regarding the use of Effective Care in Pregnancy and Childbirth[9]. The survey asked what the organizations considered the main barriers to effective transfer of research information.. Every organization specifically cited the practitioners' failure to keep up with the literature, a lack of resources, and the low value placed on research evidence as major obstructions in the implementation of research findings.

Making research information accessible and comprehensible to practitioners is an important mechanism to increasing awareness and familiarity with best clinical practices; however, it is rarely independently sufficient to overcome other categories of barriers and to effectively change practice patterns [10].

### Interventions to change professional practice

Many methods designed to change medical behavior have been developed and used in industrialized countries. Several systematic reviews on that subject concluded that there are no "magic bullets" to change professional behavior and that the best approach is to combine several strategies, such as the use of local opinion leaders, convening workshops, providing outreach visits (academic detailing), and reminders, as well as the use of audits and a provision for feedback. [11,12]. A more recent review did not support this conclusion providing evidence that some single interventions might be as effective as the combined strategies, but also acknowledging that there is a need for further and improved research regarding such interventions thus providing a strong rationale and theoretical basis for the selection of their components [13,14].

Among the trials considered in the above reviews were strategies directed at behavior change of birth attendants in North America and Europe, including distribution of educational materials, employing local opinion leaders, and the use of audits and feedback directed at increasing the number of vaginal births after cesarean section [15]; nurse opinion leaders to reduce rates of epidural analgesia by increasing the amount of support to women in labor [16]; educational outreach visits to introduce Cochrane Reviews to facilitate evidence-based use of specific birth practices [17]; employing local opinion leaders, grand round lectures, chart reminders, group interactive discussions, and audits to increase the use of corticosteroids for enhancing fetal lung maturation prior to a preterm birth [18].

Very few trials of a similar nature have been performed in developing countries, and among these results were inconclusive [19,20]. To our knowledge, no randomized controlled trial has been performed to evaluate the effectiveness of an intervention to implement evidence-based birth practices in Latin American countries.

Our hypothesis is that a multifaceted intervention designed to increase birth attendant concern about the effectiveness of routinely used birth practices, to provide them with resources and skills to access, interpret, and develop evidence-based clinical guidelines, and to establish mechanisms facilitated through key hospital leaders, to implement the guidelines and sustain them over time, will increase the use of evidence-based birth practices in Latin American hospitals. The primary objective of this trial is to evaluate the effect of this multifaceted behavioral intervention on the use of two evidence-based birth practices, the selective use of episiotomies and active management of the third stage of labor (injection of 10 IU of oxytocin). Secondary objectives are to evaluate the effect of the intervention on rates of post-partum hemorrhage, use of perineal sutures and to document readiness to change among birth attendants.

Additionally, through this process we intend to improve the research capacities of a network of Latin American hospitals, and thus increase their ability to perform quality local and collaborative research studies.

## Methods/design

**Figure 1:**
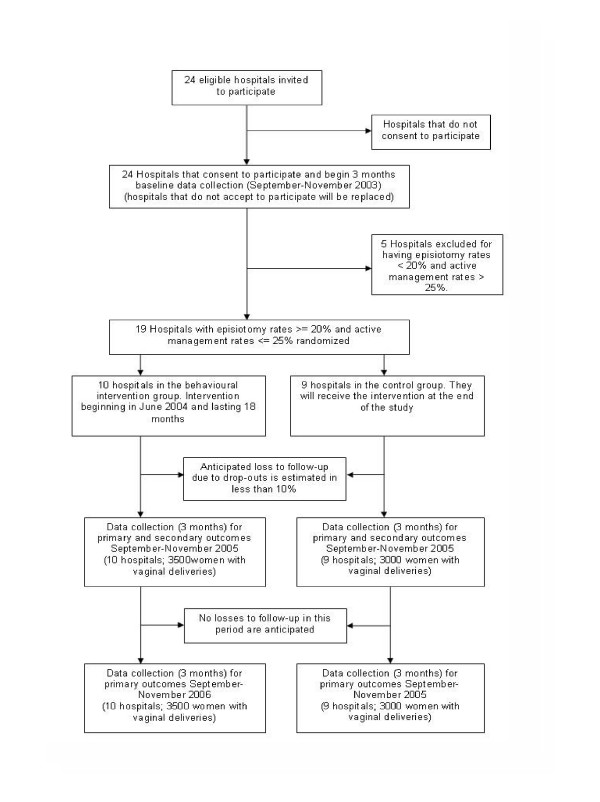
Trial Profile

The study design is a two-arm cluster randomized controlled trial with hospitals as units of randomization. Hospitals will be randomized to receive a multifaceted behavioral intervention directed at developing and implementing guidelines about episiotomy use and management of the third stage of labor, or to a control group that will continue with their usual in-service training activities. Data will be collected from eligible patients at three data collection periods, one baseline period before randomization, a post-intervention period just after the end of the intervention, and a long-term follow up one year after the end of the intervention (fig 1).

### Participating hospitals

Twenty four public hospitals in Argentina and Uruguay that had at least 1000 vaginal deliveries per year, and had no explicit policy for selective episiotomy or active management of third stage of labor were invited to participate. Those pre-selected hospitals that agreed to participate, collected baseline data. From the 24 hospitals, only 19 will be randomized because the analysis of their baseline data confirmed episiotomy rates in women with single vaginal births higher than 20%, and active management of the third stage of labor 25% or less.

### Randomization procedures

Since only 19 hospitals will participate in the intervention trial, random allocation may not provide adequate balance in the groups regarding important prognostic variables [21]. We will use a minimization procedure to assure balance between intervention and control hospitals on five important prognostic variables: 1) baseline episiotomy and active management rates, 2) residency programs, 3) country (Argentina-Uruguay), 4) hospital size (number of births per year), and 5) region within the country. Minimization has the advantage that it can match small numbers of similar units with respect to several unit characteristics [22], and may be considered methodologically equivalent to randomization [23].

Because all hospitals will enter the study at the same time, all prognostic variables are known in advance. A computer algorithm will be written to generate a large number of allocation sequences and to select only those sequences that minimize the imbalance between the two groups of hospitals. One of those sequences will be randomly selected by the computer and will be used to assign hospitals to the control or intervention groups. Both analysis of the baseline data and the allocation procedure will be done by an independent data center (Research Triangle Institute, North Carolina, USA) and their results communicated to the study coordinating unit. Thus, there will be a clear separation between the generator of the intervention allocation and the study coordination [24].

### The intervention

#### General outline

The intervention is based on the stages of change and organizational change theories and tailored by formative research [25-27]. Opinion leaders in the 12 intervention hospitals will be identified by their peers through a specific questionnaire and trained in a five day workshop to develop and implement evidence-based guidelines. They will then use a specified multifaceted approach to disseminate, implement, and maintain the guidelines in their hospitals. The 18-month intervention will include training to perform the evidence-based best practices, use of a web site, academic detailing, reminders, and feedback on utilization rates. Those components which will form the intervention are noted in Table 1 and are described in detail below.

**Table 1:** Components of the intervention

**Selection of opinion leaders**
- Teams of 3–6 birth attendants per hospital
- Selected by peer nomination
**Interactive workshops**
- Opinion leaders teams will participate in a 5 day workshop
- Objectives:
- To learn the need of an evidence based clinical practice
- To develop simple evidence based guidelines about episiotomy use and management of the third stage of labor
- To identify the barriers for the adoption of those guidelines at the hospital level
- To learn how to overcome barriers and to implement the guidelines
- To adapt and organize the dissemination and implementation of the guidelines in their hospitals
**Academic detailing**
- Dissemination of the guidelines to hospital birth attendants in small groups and individual discussions
- Identification of barriers to implement the guidelines
- Adaptation and organization of implementation activities working closely with birth attendants.
**Training on how to comply with the recommended practices**
- Training in manual abilities with videos, anatomical models and patients. One day workshop.
**Reminders**
- Placing reminders of selective episiotomy and active management of the third stage of labor in labor and delivery wards, clinical records, and surgical packages.
**Audit & feed back**
- Monthly reports of hospital episiotomy and active management rates to be distributed to every birth attendant.
**Information technology**
- Each hospital in the intervention group will receive a computer with internet access
- A specific web site will be developed to facilitate the access to study manuals and guidelines, sources of evidence-based health care literature (Reproductive Health Library, Cochrane Library), and communication among hospitals and study coordinators

#### Formative research

With the aim of refining the intervention strategy and better integrating the intervention into the hospitals' routine, a qualitative study was carried out during the preparatory phase of the trial. We conducted three in-depth interviews with upper level physician administrators (responsible for the clinical and administrative management of the departments), three focus groups with second level physicians (responsible for the individual provision of clinical care to users) and three focus groups with midwives. Additionally we conducted three focus groups with pregnant women to explore their perspectives regarding the health care they receive and their expectations of the birth practices routinely used.

#### Selection of opinion leaders

Each intervention hospital will select a team of opinion leaders who will work collaboratively during the intervention period. Each team will be composed by three to six professionals per hospital, with representation by obstetricians, residents and midwives. The department chair will be invited to designate one professional not included in the nominated team. Peer nomination will identify opinion leader team members among the professional staff of the maternity hospital using a previously validated sociometric questionnaire [28].

#### Guideline development and implementation workshops

All opinion leader teams will participate in a regional five-day workshop. There the teams will learn about the scientific basis for evidence based clinical practice, they will develop simple evidence based guidelines for episiotomy use and for the management of the third stage of labor. They will additionally suggest the main barriers for the implementation of those guidelines, and will learn and practice ways to overcome such barriers in the context of the planned intervention. Finally, each team will adapt the planned guideline dissemination and implementation activities to the characteristics of their own hospital, and will develop an organizational approach directed at the interventions.

The workshops will be given in five consecutive days, in a selected site outside the hospital walls, and will be conducted by trainers with previous experience in conducting related workshops in Latin American countries that will act as tutors, together with the country coordinators. Each tutor will guide a group with no more than 10 participants.

#### Web portal

Each hospital in the intervention group will receive a computer, access to the internet, the last issue of the WHO Reproductive Health Library [29] and access to the Cochrane Library in Spanish [30]. A study web site will be developed to facilitate the easy access to guidelines, workshop manuals, medical literature and electronic libraries and databases. This tool will be presented to the teams during the workshops, and they will later introduce the portal to each intervention hospital staff as part of the dissemination activities. The portal will be for the exclusive use of the professional staff at the intervention hospitals. A password access system will be in place during the intervention.

#### Training on manual skills

After completion of training, each opinion leader team will participate in a one-day workshop on clinical management skills (how to assist a delivery without episiotomy, and active management of the third stage of labor) using videos, anatomical models, and patients, conducted by the country coordinator or a trainer with previous experience. This workshop is based on the JHPIEGO workshop [31] currently being organized in several Latin American countries.

#### Dissemination of guidelines at the hospital and identification of barriers

Each opinion leader team disseminates the guidelines among the professional staff at its respective hospitals. They will primarily utilize academic detailing as the strategy for this purpose [32]. The team will present and discuss the guidelines with all birth attendants, providing to each one the opportunity to discuss them and to identify specific barriers; the birth attendants will adapt the planned activities for overcoming the barriers and develop an implementation timetable. Finally, the team will present the web portal, and will select a group of potential early adopters (volunteers) who appear enthusiastic for participation in the implementation of the guidelines.

#### Implementation and maintenance of guidelines

The objective of this component is to facilitate deliveries by birth attendants according to the recommendations developed in the workshops. Three strategies will be carried out to achieve this aim:

a) Training on how to do the recommended practices: all birth attendants will receive one training session given by the opinion leaders, on anatomical models and on patients.

*b*) Placing reminders: there will be short messages that remind birth attendants to consider the two evidence-based birth practices of interest:

• Active management reminders: to be placed in the partograph and as posters in the delivery ward.

• Selective episiotomy reminders: to be placed on the surgical package for assisting delivery and in the partograph.

The text of the reminders will be prepared by the opinion leader teams according to the designed recommendation and will take into account the characteristics and barriers of each specific hospital.

c) Monthly reporting of the episiotomy rates and the use of oxytocin for management of the third stage of labor at the hospital level: This information will be produced by their Perinatal Information System (PIS) [33] and distributed to birth attendants.

The intervention will be pilot tested in two hospitals similar to the hospitals that will participate in the trial.

### Activities in control hospitals

Birth attendants from hospitals in the control group will receive no intervention after randomization other than their standard in-service training activities and standard sources of information. We intend to provide birth attendants with the guideline development component, a computer and bibliography *following *the completion of the study.

### Outcome measures

The primary outcomes are aimed at changing the birth attendants' behavior relative to the rate of episiotomy and the rate of injection use of 10 I.U. of oxytocin during third stage of labor. Secondary clinical outcomes will be studied to estimate the health impact of the intervention, including the rate of perineal sutures use and the incidence of postpartum hemorrhage >500 ml. These outcomes will be assessed in singleton vaginal deliveries. We will also measure the provider's readiness to change with respect to episiotomies and management of third stage of labor with a specific questionnaire.

All outcomes will be assessed for a three-month period at three time points: at baseline (before randomization), after the end of the intervention, and the primary outcomes one year after the end of the intervention to measure the long term effects of the intervention. Interim analysis will be performed as required by the Data and Safety Monitoring Committee.

### Process measures

Process data will be collected during the intervention at the active intervention hospitals, with the objective of allowing for early detection and correction of implementation problems. This will allow detection of implementation problems if the intervention is not effective or facilitation of the intervention if it is effective. Three categories of process measures will be considered: program inputs, guideline implementation activities, and birth attendants' reactions.

### Data collection

Although the hospital will be the unit of analysis, we shall collect individual patient data in order to assure data quality. The goal is to accumulate data on approximately 300–500 deliveries from each hospital. All the hospitals in the project use a standard perinatal clinical record form (PIS form) [33]. This form includes the data of the obstetric history, prenatal care, labor, delivery, and neonatal outcomes. To obtain the additional necessary data to calculate the outcome variables, we will implement a modified version of the PIS clinical record that will add some variables at the bottom of the form to be addressed during the data collection periods. This approach will reduce to a minimum extra activities associated with data collection by the birth attendants during the study.

Blood loss during the third stage of labor (after delivery) will be measured from all vaginal deliveries during the data collection periods. A plastic, transparent, collection drape will be employed. At the end of the blood collection period, the amount of blood loss will be measured by pouring the contents of the drape into a calibrated pitcher. The amount of blood loss will be recorded in the data form together with the time (beginning and end) of the blood collection.

To measure provider's readiness to change, a self-administered questionnaire was designed and will be completed by all physicians and midwives at all participating hospitals, before randomization and at the end of the intervention.

### Data management

The data collection system to determine outcomes will be independent from the implementation of the intervention. Given the nature of the intervention, we cannot blind the randomization, and data collectors will likely know if they are part of an intervention/control hospital. In addition, intervention hospitals will monitor and record clinical data (i.e., episiotomy rate and active management of the third stage of labor) through their routine data collection system. As a consequence, it is expected that the proposed intervention will improve the capacity of intervention hospitals to collect and review clinical data with the potential of introducing bias in the outcome assessment. To minimize this bias, the data collection system will as much as possible be isolated from the intervention instruments.. In practical terms, this means that data collectors at the hospital level, the data supervisors and the associated computer software used for data collection will not be associated with intervention activities.

Each participating hospital will receive a computer with an Internet access to be used for data collection purposes. A custom developed data management software program will be installed in each computer and will be used by trained and certified in-hospital data collectors, for distributed data entry, data validation and data transmission to the coordinating unit. The system provides a secure environment for confidential medical information and the access to it will be limited to authorized individuals. No personal identifiers will be transmitted from the hospital to the data coordinating unit

### Sample size

The sample size was estimated based upon the hospital being the unit of analysis. A preliminary analysis of data from Argentinean hospitals [2] documented a baseline frequency of episiotomies in vaginal deliveries of 42%, with a standard deviation of 11%. To protect against changes in the standard deviation [15], we have based our calculations on a standard deviation of 15%. Thus, we need 18 hospitals (9 intervention and 9 control) to identify a decrease of episiotomy rates from 40% to 20%, with a 0.05 significance level and 80% power. Considering that in public hospitals in Argentina and Uruguay about 25% of deliveries are seen among primiparae, and 75% among multiparae, a reduction of the general episiotomy rate from 40% to 20% could be achieved by reducing the rate among primiparae from 80% to 40%, and the rate among multiparae from 26% to 13%. The rates after this intervention would thus be similar to the rates achieved in a previous trial [34] and are therefore potentially attainable. Moreover, the sample size of 18 hospitals will allow identification of smaller decreases of episiotomy rates among primiparae vaginal deliveries, from 80% to 60%, with a 0.05 significance level and 80% power.

We expect the use of oxytocin during the third stage of labor to increase from 10% to 50%. However, assuming a baseline frequency of oxytocin use of 10% and a standard deviation of 5%, a sample size of 18 hospitals will provide a power > 95% to identify an increase in oxytocin use from 10% to 20%, with a 0.05 significance level. Assuming a baseline incidence rate of post-partum hemorrhage of 15% and a standard deviation of 5%, a sample size of 18 hospitals will provide a power of 84% to identify a reduction in hemorrhage from 15% to 8%.

Hospitals will initiate baseline data collection to obtain information that will be used to assess the inclusion criteria before randomization. A total of 24 hospitals were included to allow for hospital drop-outs.

### Data analysis

Inference will be primaryly directed at the cluster (hospital) level. All analyses addressing the study research questions will use the "intention to treat" principle, comparing the outcome in the intervention group to the nonintervention group.

The outcome variables will be the percentage of primary and secondary outcomes measured during the three months following the end of the intervention. Mantel Haenszel summary risk ratios combining the individual ratios for each hospital in the intervention group and non-intervention group will be computed. The intervention and non-intervention groups will then be compared at the group level using Student's t-test on the logarithms of the summary risk ratio [17].

In addition, the data collected at the individual level will be used to explore the potential for confounding of the main effects of the intervention due to imbalances arising from the group randomization. Such multi-level analyses will use mixed model techniques (hierarchical linear models) [35,36].

For the analysis of provider's "readiness to change," the data will be summarized in terms of medians and interquartile ranges. Non-parametric statistics will be used to compare the pre- and post-intervention differences between the control and intervention groups.

### Ethical aspects

This is a cluster randomized trial with an intervention targeted at the cluster (hospital) level; the behavioral intervention will be directed to the entire group of birth attendants at each intervention hospital. Given that the purpose of the study is to improve the quality of care at the hospitals, this should not imply any additional risk for women and their children. Birth attendants are the subjects of the trial, although the main outcomes will be measured in terms of the patient. All responsible hospital authorities are to be provided written agreements to participate in advance of randomization [37,38]. Birth attendants in the intervention hospitals will receive a fact sheet with information as to the format, length, and purpose of the training intervention. In addition, birth attendants who are nominated as opinion leaders in the intervention hospitals will also provide written agreements to participate in that role.

The necessary data for outcome measurement is routinely collected at the hospitals, and no personal identifiers will be transmitted with in the data to the coordinating unit; thus there will be need no need to obtain informed consent from patients.

All birth attendants will provide written consent before filling out the 'Readiness to Change' questionnaire and all measures will be taken to keep their identities confidential

The protocol was submitted and approved by the IRBs of the following institutions: University of North Carolina at Chapel Hill; Tulane University; Research Triangle Institute; Pan American Health Organization; School of Medicine of the University of the Republic in Uruguay; the University Hospital of Montevideo, Uruguay; and the Argentinean Society for Clinical Research.

The trial is registered at the NIH clinical trials register (; Identifier: .NCT00070720) and at the Current Controlled Trials Register ; ISRCTN82417627).

## Abbreviations

IU: international units

WHO: World Health Organization

PIS: Perinatal Information System

IRB: Institutional review board

NIH: National Institutes of Health

NICHD: National Institute of Child Health and Human Development

## Competing interests

The author(s) declare that they have no have competing interests.

## Authors' contributions

P Buekens, J Belizán and F Althabe had the original idea and designed the intervention and the first protocol. F Althabe, E Bergel, P Buekens, and J Belizán wrote the final protocol, in collaboration with N Kropp, L Wright, N Goco, N Moss, and the rest of the Guidelines Trial Group.

## Appendix 1 - Guidelines Trial Group

**Principal Investigators: **P Buekens, JM Belizán

**Trial Coordinating Unit**: J Belizán (Principal Investigator), F Althabe (Trial Coordinator), E Bergel (Statistician and Data Coordinator), M Delgado (Programmer), A Ciganda (Data Manager), G Tomasso (Intervention Manager), A Codazzi (Research Assistant), M Colomar (Research Assistant).

**Intervention development team**: P Buekens, JM Belizán, F Althabe, M Campbell, G Sotero, G Tomasso, ML Cafferata, B Dugan, S Cohen.

**Data management system design team: **E Bergel, M Delgado, A Ciganda

**Statistical support**: E Bergel, S Bandiwala, Q Yao, T Hartwell, H Chakraborty

Countries' Coordinating teams:

**Argentina**: A Blake, A Karolinski (Country Coordinators); AM Bonotti, A del Pino, A Sánchez, M Walker (Data Supervisors).

**Uruguay**: G Sotero (Country Coordinator), A Ciganda (Data Supervisor).

**Formative research team: **M Belizán, M Campbell, A Codazzi, M Colomar, A Meier.

**Global Network for Women's and Children's Health Research supporting team: **LWright (Director), T Hartwell (PI Data Center), N Moss (Program Officer), N Kropp (Protocol Manager), N Goco (Former Protocol Manager).

## Pre-publication history

The pre-publication history for this paper can be accessed here:


